# Supplementary material for: Adaptive Pruning for Increased Robustness and Reduced Computational Overhead in Gaussian Process Accelerated Saddle Point Searches
Source: Chemphyschem. 2026 Feb 23;27(4):e202500730. doi: 10.1002/cphc.202500730 (PMC12927439; doi:10.1002/cphc.202500730)
Supplement: Supplementary file 1 — Supplementary Material [file CPHC-27-e202500730-s001.pdf]

Adaptive Pruning for Increased Robustness and Reduced Computational Overhead in Gaussian Process Accelerated Saddle Point Searches

Supplementary Material

Rohit Goswami[1,2] and Hannes Jónsson[1]

[1]Science Institute, University of Iceland, Reykjavik, Iceland
[2]Laboratory of Computational Science and Modeling, Institute of Materials, École Polytechnique Fédérale de Lausanne, 1015 Lausanne, Switzerland

November 17, 2025

Contents

S1 Reproduction note **S2**

S2 Rotation removal implementation **S2**

S3 More computational details **S3**

S4 Distance measure quantification **S4**
S4.1 Notational clarity S5

S5 Fragment detection **S5**

S6 D136 and the effect of initial conditions **S5**
S6.1 Initialization S5
S6.2 Post-Initialization Comparability for D136 . . . . . S6

S7 Statistical analysis of cost **S8**
S7.1 Model specifications S8
S7.1.1 Prior distributions S8
S7.1.2 Computational settings S9
S7.1.3 Number of PES calls S9
S7.1.4 Total wall-clock time S10

S8 GPDimer / OTGPD failure modes **S13**
S8.1 D016 . . . . . S15
S8.2 D084 . . . . . S15

S8.3 D100 . . . . S15
S8.4 S242 . . . . S15

Code for reproduction on Github. Data on Materials Archive

Github `https://github.com/TheochemUI/otgpd_repro`

Materials Archive `https://doi.org/10.24435/materialscloud:rh-tw`

S1 Reproduction note

The full set of benchmark inputs, raw outputs, analysis scripts, and pinned runtime environments used in this study are publicly archived. The GitHub repository and Materials Cloud archive contain the original runs for all 238 systems, the scripts used to generate every figure and table, and environment specifications (container/environment manifests) that reproduce the computational environment.

To diagnose a small number of non-convergent cases we performed local re-runs only for the four systems which fail for the OTGPD but not the GPDimer; these runs and their logs are included in the same archive and flagged in the repository for convenience. Users wishing to reproduce any specific experiment can either use the provided raw outputs, the pre-processed FAIR formatted `csv` data, or re-run the workflow using the included environment manifests and Snakemake pipelines; exact instructions and file paths are given in the archive and on the Github repository.

S2 Rotation removal implementation

Our Gaussian Process model approximates the potential energy surface without inherent knowledge of the physical invariances of the system. Consequently, a proposed optimization step may contain spurious components corresponding to the external degrees of freedom: overall translation and rotation of the entire molecule. The optimizer actively removes these components from the proposed translation step vector to ensure that movements occur only along internal coordinates, which represent genuine changes in molecular geometry.

The procedure first constructs a basis set spanning the space of infinitesimal rigid-body motions. For a system of N atoms, this space has six dimensions (or five for a linear molecule). The procedure generates three basis vectors for translation, $\{\mathbf{t}_x, \mathbf{t}_y, \mathbf{t}_z\}$, where each vector $\mathbf{t}_k$ represents a unit displacement of all atoms along the Cartesian axis k.

$$(\mathbf{t}_k)_{3i+k-1} = 1 \quad \forall i \in \{1, ..., N\} \tag{1}$$

Next, the procedure generates three basis vectors for rotation, $\{\mathbf{l}_x, \mathbf{l}_y, \mathbf{l}_z\}$, derived from the expression for infinitesimal rotation about the center of mass, $\mathbf{r}'_i = \mathbf{r}_i - \mathbf{r}_{\text{com}}$. An infinitesimal rotation of the entire system corresponds to a displacement $\delta\mathbf{r}_i = \delta\omega \times \mathbf{r}'_i$. The rotational basis vectors thus take the form:

$$\mathbf{l}_x = \sum_{i=1}^{N} \hat{\mathbf{e}}_x \times \mathbf{r}_i' \tag{2}$$

$$\mathbf{l}_y = \sum_{i=1}^{N} \hat{\mathbf{e}}_y \times \mathbf{r}_i' \tag{3}$$

$$\mathbf{l}_z = \sum_{i=1}^{N} \hat{\mathbf{e}}_z \times \mathbf{r}_i' \tag{4}$$

The algorithm then applies the Gram-Schmidt process to this set of six vectors to produce an orthonormal basis, $\{\mathbf{u}_k\}$, that spans the external degrees of freedom. For any proposed translation step, $\mathbf{s} \in \mathbb{R}^{3N}$, the algorithm projects out the external components. The component of the step corresponding to translation and rotation, $\mathbf{s}_{\text{ext}}$, projects onto this basis:

$$\mathbf{s}_{\text{ext}} = \sum_{k} (\mathbf{s} \cdot \mathbf{u}_k)\mathbf{u}_k \tag{5}$$

The pure internal step, $\mathbf{s}_{\text{int}}$, then becomes the original step minus its external projection:

$$\mathbf{s}_{\text{int}} = \mathbf{s} - \mathbf{s}_{\text{ext}} \tag{6}$$

A feedback mechanism enhances the stability of the GP-driven search. The algorithm computes the magnitude of the removed component, $\|\mathbf{s}_{\text{ext}}\|$. If this magnitude exceeds a defined threshold, θ_{rot}, it signals that the GP model likely predicts a large, unphysical torque on the molecule. In such cases, the procedure discards the projection and reverts to the original, unprojected step $\mathbf{s}$. Subsequent step-size limitation guardrails then typically intercept this large, physically questionable step, triggering a resampling of the true potential energy surface to improve the GP model. When the magnitude of the removed component remains below the threshold, the algorithm accepts the purified internal step $\mathbf{s}_{\text{int}}$. This ensures a more precise update to the molecular geometry, guided only by genuine internal forces.

In practice, since energy doesn't depend on rotations, the threshold tends to large values.

S3 More computational details

The analysis was performed on a dataset of 500 initial configurations of small organic molecules with between 7 and 25 atoms. All versions of software used with the exception of NWChem are vendored.

- For EON[1], >2.8.0 should be compatible
- NWChem[2] integration requires this pull request allowing clients to poll for inputs

Searches were aborted if they exceeded 1000 iterations, if the energy increased by more than 20 eV, or if NWChem produced a fatal error. The final convergence criterion for a saddle point was a root-mean-square force below 0.01 eV/Å.

S4 Distance measure quantification

The core of the problem is that the inverse distance we use is not invariant to the permutation of identical atoms, and since kernel's value depends on a direct, index-wise comparison of the interatomic distance vectors of two configurations. This creates a dependency on the arbitrary, fixed labels of the atoms, rather than their physical roles.

An easy way to understand this stems from observing symmetric systems. For instance, consider a proton (indexed k) transferring between two chemically equivalent sites (m and n). Physically, the initial and final states are energetically degenerate. However, a fixed-index comparison metric perceives a significant geometric change, as the distance r(k,m) transitions from short to long, while r(k,n) simultaneously transitions from long to short. The metric fails to recognize that the permutation of labels would reconcile the apparent structural difference.

While the kernel's fitted length-scale hyperparameter may partially average out this effect, a non-averaged metric for early stopping feels the full impact of the flaw. The 1D max log distance, by its definition, registers a significant, non-physical distance for this symmetric swap:

$$D_{\text{1Dmaxlog}}(\mathbf{x}_1, \mathbf{x}_2) = \max_{i,j} \left| \log \frac{r_{ij}(\mathbf{x}_2)}{r_{ij}(\mathbf{x}_1)} \right|$$

This sensitivity to labeling motivates using the intensive EMD. Figure S1 demonstrates this, by contrasting the behavior of both metrics for the asymmetric stretching of a water molecule.

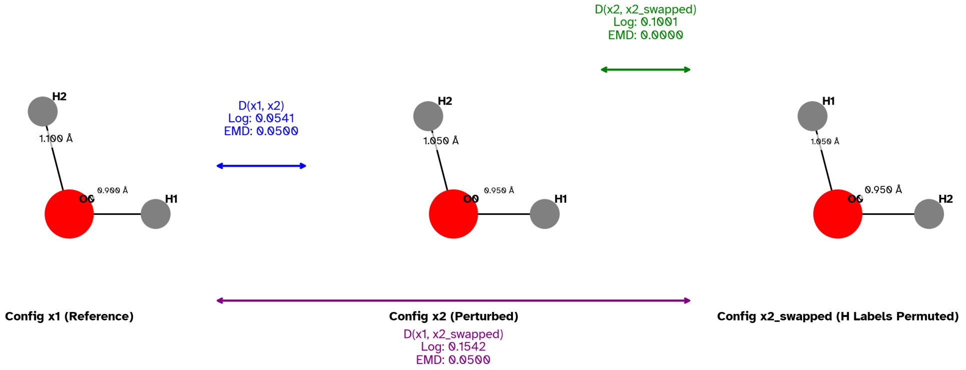

Figure S1: Comparison of the 1D max log distance and the Earth Mover's Distance (EMD) for an asymmetric stretch of a water molecule. While configuration x_2 and $x_{2,s}$ are physically identical (differing only by the permutation of hydrogen atom labels), the 1D max log metric incorrectly assigns a large distance between them and the reference x_1. In contrast, the EMD correctly identifies them as being equidistant from the reference, demonstrating its permutational invariance.

S4.1 Notational clarity

An inequality expresses the measure in the literature[3]:

$$\frac{2}{3}r_{ij}(\mathbf{x}_{\text{eval}}) < r_{ij}(\mathbf{x}_{\text{im}}) < \frac{3}{2}r_{ij}(\mathbf{x}_{\text{eval}})$$

This is however equivalent.

$$\left|\log\frac{r_{ij}(\mathbf{x}_{\text{im}})}{r_{ij}(\mathbf{x}_{\text{eval}})}\right| < \log(1.5) \approx 0.405$$

S5 Fragment detection

To unambiguously distinguish covalently bonded molecular fragments from transient non-covalent contacts within the simulation cell, a quantum mechanical bonding analysis was performed. For each system configuration, the Mayer-Wiberg-type bond orders for all atom pairs were calculated using the GFN2-xTB semi-empirical method[4], as implemented in the `tblite` library.

Each calculation was performed on the entire supercell. The total charge of the system was set to zero, and the spin multiplicity was explicitly defined as either singlet (1 unpaired electron pair) or doublet (1 unpaired electron), corresponding to the pre-determined electronic state of the system.

From the resulting bond order matrix, a molecular connectivity graph was constructed. A chemical bond between two atoms, i and j, was defined to exist if and only if their calculated bond order, BO_ij, exceeded a threshold, 0.7 here. Based on a calibration procedure for representative configurations, a bond order threshold of 0.7 was selected. This value provides a robust margin to exclude minor, non-zero bond orders (up to ≈ 0.56) calculated for close inter-fragment contacts, while safely including all genuine covalent bonds (typically with BO_{ij} > 0.9). Finally, the distinct molecular fragments were identified by determining the connected components of the resulting graph. Fragments with geometric centers less than 1 angstrom away were merged together to take into account slightly fragmented systems.

S6 D136 and the effect of initial conditions

This section provides detailed evidence supporting the direct comparison of the OTGPD and GPDimer methods in the main text. The data demonstrate that the initial rotation phase on the true potential energy surface effectively equalizes the starting point for the main search, irrespective of the initial guess heuristic.

S6.1 Initialization

The two methods begin with different procedures to generate the initial atomic displacement.

OTGPD we implemented a new displacement heuristic in the EON code. This method displaces the structure by a fixed magnitude of 0.01 Å along the softest vibrational mode. An inexpensive GFN2-xTB semi-empirical calculation determines this mode, and the displacement only affects atoms not designated as frozen.

GPDimer[?] we utilize a standard displacement heuristic pre-existing in the EON code. This method follows a multi-step stochastic process: first, it identifies the least coordinated atom(s) using a 3.3 Å cutoff radius. It then randomly selects one of these atoms as a displacement epicenter and displaces it along with all neighboring atoms within a 5.0 Å radius. The displacement for each atom follows a 3D vector drawn from a Gaussian distribution, where the 0.01 Å `displace_magnitude` parameter serves as the standard deviation.

S6.2 Post-Initialization Comparability for D136

We analyze the initial conditions and saddles from a structural perspective using the Constrained Shortest Distance Assignments (CShDA)[5].

```
cd runs
python init_condcheck.py automated/snake_runs/softest_mode_scg_barrier/doublet/136 \
    ../../gprd_sella_bench/bench_runs/eon/runs/1e8m/gprd/final_gprd_wparam/doublets/136/
```

```
                          Structural & Vector Comparison
+-------------------------------------+-------------------------------------+
|                              Metric | Value                               |
+-------------------------------------+-------------------------------------+
|                     Run 1 Directory | softest_mode_scg_barrier/doublet/136 |
|                     Run 2 Directory | gprd/final_gprd_wparam/doublets/136  |
|-------------------------------------+-------------------------------------|
|      Start Structure CSHDA RMSD (Å) | 0.000000                            |
|  Displaced Structure CSHDA RMSD (Å) | 0.004925                            |
|     Saddle Structure CSHDA RMSD (Å) | 0.007412                            |
|        Direction Cosine Similarity | 0.008106                            |
+-------------------------------------+-------------------------------------+
           INFO     Comparing results.dat metrics...
                          results.dat Comparison
+----------------------------+---------------+---------------+------------+
|                     Metric |         Run 1 |         Run 2 | Difference |
+----------------------------+---------------+---------------+------------+
|          final_eigenvalue |      -42.1910 |      -39.8012 |      +2.39 |
|         force_calls_saddle |            26 |            38 |        +12 |
|                 iterations |            23 |            34 |        +11 |
|                   job_type | saddle_search | saddle_search |          - |
|  potential_energy_reactant |    -9430.1098 |    -9430.1097 |   +7.7e-05 |
|    potential_energy_saddle |    -9431.1361 |    -9431.1361 |     -2e-06 |
|             potential_type |  SocketNWChem |    ASE_NWCHEM |          - |
```

```
|             random_seed |         1995 |    706253457 | +706251462 |
|      termination_reason |      Success |      Success |          - |
|       total_force_calls |           28 |           39 |        +11 |
+-------------------------+--------------+--------------+------------+

Notes:
- CSHDA RMSD closer to 0 means more similar structures (permutation-invariant).
- Cosine Similarity closer to 1 means more similar directions (0=orthogonal, -1=opposite).
```

The two heuristics produced nearly orthogonal initial displacement vectors (cosine similarity ≈ 0.01). However, the subsequent rotation on the true PES, a process common to both methods, which takes up to 6 iterations on the true energy surface, effectively erases this initial difference. This equalization procedure yields nearly identical final saddle structures (RMSD ≈ 0.007 Å) and energies. Consider, also the log for the hyperparameters after the initial rotation:

```
# GP Dimer
Rotated the initial dimer in 4 outer iterations (total number of image evaluations: 5).
# Hyperparameters
magnSigma2: 0.0304955
lengthScales:
0.102959
0.239712
0.170075
0.235037
0.718695
# Total time
[2025-09-23T09:41:55Z TRACE] pixi r eonclient took 19m 58s 574ms 382us 392ns to complete.
# OTGP-Dimer
Rotated the initial dimer in 2 outer iterations (total number of image evaluations: 3).
# Hyperparameters
magnSigma2: 0.0634641
lengthScales:
0.102861
0.417811
0.161203
 0.58395
0.834778
# Total time
[2025-02-03T03:35:33Z TRACE] pixi r eonclient took 45m 47s 580ms 955us 824ns to complete.
```

This show that, the initial hyperparameters learned by each GP model show significant similarity, as expected for models observing the same local potential energy surface. The differences stem from SCG barrier method which changes the likelihood of the OTGP as discussed in the main text. The OTGP-Dimer method shows larger length scales (l_2, l_4, l_5) compared to GP Dimer. These larger values indicate that the OTGPD model perceives the potential energy surface as smoother

along these specific coordinates. Collectively, this demonstrates that the main search phase for both algorithms begins from a physically equivalent and directly comparable state.

S7 Statistical analysis of cost

Two hierarchical Bayesian regression models were fitted to quantify the impact of the three search algorithms (OT-GPD, GP-Dimer, standard Dimer) on (i) the number of expensive potential-energy-surface (PES) evaluations and (ii) the total wall-clock time required to locate a first-order saddle point. Both models share the same random-effect structure (a molecule-specific intercept that also accounts for spin multiplicity) and were implemented in the `brms` R package (v2.19.1) using the `cmdstanr` backend.

We fit hierarchical Bayesian regression models (negative-binomial for the number of PES evaluations and Gamma for the wall-clock time) with the method (OT-GPD, GP-Dimer, standard Dimer) as a fixed effect and a random intercept for each molecule–spin combination[6]. We report posterior medians together with 95% credible intervals (CrI) in Tbl. S1.

Table S1: Table of modeled performance metrics

Method	Median PES Calls	Time (min)	95% CrI Time
OTGPD	28	9.0	[8.6, 9.5]
Dimer	254	20.5	[19.2, 21.8]
GPDimer	30	12.8	[12.2, 13.5]

The statistical model confirms the practical gains of the OT-GP framework. Compared with the baseline Dimer, OTGPD achieves an $\approx$ 89% reduction in the median number of expensive force evaluations (254 → 28 calls) and more than halves the median time-to-solution (20.5 min → 9.0 min). The improvement over the earlier GP-Dimer also proves decisive: the OTGPD runs $\approx$ 30% faster (9.0 min versus 12.8 min) while using slightly fewer force calls. The posterior distributions for these effects show clear separation from zero, indicating a >99% probability that OTGPD outperforms both comparators.

We may use such models for interpretation, understanding that these provide additional insights into the distributional details of the raw data.

S7.1 Model specifications

S7.1.1 Prior distributions

We use weakly informative priors, chosen to regularise the models without imposing strong beliefs about the magnitudes of effects, detailed in Table S2.

We specified priors identically for both models, the `exponential(1)` prior for scale/shape parameters has a mean of 1, reflecting a modest expectation of dispersion.

Table S2: Priors for predictive model

Parameter	Prior (distribution)	Rationale
Fixed-effect coefficients (β_{method}, γ_{method})	`normal(0, 1)`	Allows a 95 percent prior range of roughly ± 2 on the log scale ($\approx e^{-2}$ to e² ≈ 0.14–7.4), comfortably covering plausible speed-up or slow-down factors.
Intercept (β_o, γ_o)	`student_t(3, 0, 2.5)`	Heavy-tailed to tolerate outliers while still centring near zero.
Random-effect standard deviation ($\sigma_m ol$)	`exponential(1)`	Strongly shrinks the group variance toward zero unless data demand otherwise.
Shape parameter of the negative-binomial (Ψ)	`exponential(1)`	Enforces positivity and encourages modest over-dispersion.
Shape parameter of the Gamma (α)	`exponential(1)`	Guarantees positivity and avoids overly heavy tails.

S7.1.2 Computational settings

Both models were fitted with four parallel Markov chains, each with 4 000 iterations (the first 1 000 discarded as warm-up). The total effective sample size for all parameters exceeded 1 200 ($\geq 3\times$ the number of chains) and the potential scale reduction factor ($\widehat{R}$) was $\leq$ 1.01 for every parameter, indicating convergence. Sampling was performed with the NUTS (No-U-Turn Sampler) algorithm implemented in CmdStan (v2.35.0) via the `cmdstanr` R interface. To ensure stable Hamiltonian dynamics we set `adapt_delta = 0.99` and `max_treedepth = 15` for the wall-time model (the negative-binomial model converged with the default settings).

S7.1.3 Number of PES calls

The count data may be over-dispersed relative to a Poisson distribution, therefore we employ a negative-binomial likelihood (log link):

$$
\begin{aligned}
\text{pes\_calls}_i &\sim \text{NegBinomial}(\mu_i, \phi) \\
\log(\mu_i) &= \beta_0 + \beta_{\text{method}[i]} + u_{j[i]} \\
u_j &\sim \mathcal{N}(0, \sigma^2_{\text{mol}}) \quad (j = \text{mol\_id:spin}).
\end{aligned}
$$

- *Fixed effects* (β_{method}) encode the multiplicative shift relative to the baseline Dimer (reference level).
- *Random intercept* (u_j) captures systematic differences among molecules and between spin states.

```
Family: negbinomial
 Links: mu = log
```

```
Formula: pes_calls ~ method + (1 | mol_id:spin)
   Data: data (Number of observations: 692)
  Draws: 4 chains, each with iter = 4000; warmup = 1000; thin = 1;
         total post-warmup draws = 12000

Multilevel Hyperparameters:
~mol_id:spin (Number of levels: 238)
              Estimate Est.Error l-95% CI u-95% CI Rhat Bulk_ESS Tail_ESS
sd(Intercept)     0.35      0.02     0.31     0.40 1.00     4042     7082

Regression Coefficients:
              Estimate Est.Error l-95% CI u-95% CI Rhat Bulk_ESS Tail_ESS
Intercept         5.61      0.03     5.55     5.67 1.00     6816     7994
methodGPDimer    -2.16      0.03    -2.22    -2.10 1.00    17086     9663
methodOTGPD      -2.26      0.03    -2.32    -2.20 1.00    16874     9285

Further Distributional Parameters:
      Estimate Est.Error l-95% CI u-95% CI Rhat Bulk_ESS Tail_ESS
shape    11.26      0.87     9.65    13.03 1.00     7688     8984

Draws were sampled using sample(hmc). For each parameter, Bulk_ESS
and Tail_ESS are effective sample size measures, and Rhat is the potential
scale reduction factor on split chains (at convergence, Rhat = 1).
```

With the exact results in Table S3.

Table S3: Results of the PES model

$\text{Effect}_{\text{Type}}$	Median Effect	95% CrI
Expected PES Calls (Baseline: Dimer)	272.8	[256.7, 289.9]
Multiplicative Factor (GPDimer vs Dimer)	0.1	[0.11, 0.12]
Percentage Change (GPDimer vs Dimer)	-88.4%	[-89.1%, -87.7%]
Multiplicative Factor (OTGPD vs Dimer)	0.1	[0.10, 0.11]
Percentage Change (OTGPD vs Dimer)	-89.6%	[-90.2%, -88.9%]
sd(Intercept) [mol_{id}:spin]	0.3	[0.31, 0.40]

S7.1.4 Total wall-clock time

Since wall-clock times follow a continuous, strictly positive, and right-skewed distribution, we use a Gamma likelihood with a log link. To capture the complex, non-linear relationship between the number of PES evaluations and the total time, which can differ significantly between methods due to varying overhead costs, we employ a generalized additive model (GAM). Specifically, we model the log of the expected time as a function of method-specific smoothing splines of the log-transformed PES calls:

$$\text{tot\_time}_i \sim \text{Gamma}(\alpha, \beta_i)$$
$$\log(\beta_i) = \gamma_0 + \gamma_{\text{method}[i]} + s_{\text{method}[i]}(\log(\text{pes\_calls}_i)) + v_{j[i]}$$
$$v_j \sim \mathcal{N}(0, \sigma^2_{\text{mol}}).$$

In this formulation, the term $s_{\text{method}[i]}(\cdot)$ represents a unique thin-plate regression spline for each method, allowing the model to learn the distinct, non-linear time-cost profiles. The fixed effects (γ_{method}) capture the baseline differences between methods, while the random intercept (v_j) accounts for system-specific variations, as in the PES calls model. This more flexible structure allows for a more accurate and nuanced comparison of method efficiencies across their operational ranges.

```
 Family: gamma
  Links: mu = log
Formula: tot_time ~ method + s(log_pes_calls, by = method, k = 5) + (1 | mol_id:spin)
   Data: data (Number of observations: 1433)
  Draws: 4 chains, each with iter = 4000; warmup = 1000; thin = 1;
         total post-warmup draws = 12000

Smoothing Spline Hyperparameters:
                                   Estimate
sds(slog_pes_callsmethodDimer_1)       2.44
sds(slog_pes_callsmethodGPDimer_1)     3.79
sds(slog_pes_callsmethodOTGPD_1)       4.15
                                   Est.Error
sds(slog_pes_callsmethodDimer_1)        0.51
sds(slog_pes_callsmethodGPDimer_1)      0.52
sds(slog_pes_callsmethodOTGPD_1)        0.51
                                   l-95% CI
sds(slog_pes_callsmethodDimer_1)       1.56
sds(slog_pes_callsmethodGPDimer_1)     2.87
sds(slog_pes_callsmethodOTGPD_1)       3.22
                                   u-95% CI Rhat
sds(slog_pes_callsmethodDimer_1)       3.53 1.00
sds(slog_pes_callsmethodGPDimer_1)     4.89 1.00
sds(slog_pes_callsmethodOTGPD_1)       5.24 1.00
                                   Bulk_ESS
sds(slog_pes_callsmethodDimer_1)       8533
sds(slog_pes_callsmethodGPDimer_1)    10689
sds(slog_pes_callsmethodOTGPD_1)      12370
                                   Tail_ESS
sds(slog_pes_callsmethodDimer_1)       6729
sds(slog_pes_callsmethodGPDimer_1)     7913
sds(slog_pes_callsmethodOTGPD_1)       8356
```

```
Multilevel Hyperparameters:
~mol_id:spin (Number of levels: 499)
              Estimate Est.Error l-95% CI u-95% CI
sd(Intercept)     0.39      0.02     0.35     0.42
              Rhat Bulk_ESS Tail_ESS
sd(Intercept) 1.00     3036     6241

Regression Coefficients:
                               Estimate Est.Error
Intercept                          2.71      0.12
methodGPDimer                      1.15      0.22
methodOTGPD                       -0.11      0.28
slog_pes_calls:methodDimer_1       1.51      0.95
slog_pes_calls:methodGPDimer_1     0.44      1.00
slog_pes_calls:methodOTGPD_1       0.22      1.00
                               l-95% CI u-95% CI
Intercept                          2.48     2.94
methodGPDimer                      0.72     1.58
methodOTGPD                       -0.67     0.44
slog_pes_calls:methodDimer_1      -0.33     3.38
slog_pes_calls:methodGPDimer_1    -1.54     2.40
slog_pes_calls:methodOTGPD_1      -1.74     2.16
                               Rhat Bulk_ESS
Intercept                      1.00    10213
methodGPDimer                  1.00     7315
methodOTGPD                    1.00     7428
slog_pes_calls:methodDimer_1   1.00    13657
slog_pes_calls:methodGPDimer_1 1.00    19482
slog_pes_calls:methodOTGPD_1   1.00    19934
                               Tail_ESS
Intercept                          9335
methodGPDimer                      8663
methodOTGPD                        7717
slog_pes_calls:methodDimer_1       9152
slog_pes_calls:methodGPDimer_1     8434
slog_pes_calls:methodOTGPD_1       9243

Further Distributional Parameters:
      Estimate Est.Error l-95% CI u-95% CI Rhat
shape     7.73      0.36     7.04     8.47 1.00
      Bulk_ESS Tail_ESS
shape     6529     8211

Draws were sampled using sample(hmc). For each parameter, Bulk_ESS
```

and Tail_ESS are effective sample size measures, and Rhat is the potential scale reduction factor on split chains (at convergence, Rhat = 1).

We show the model predictions overlaid with the data in Figure S2.

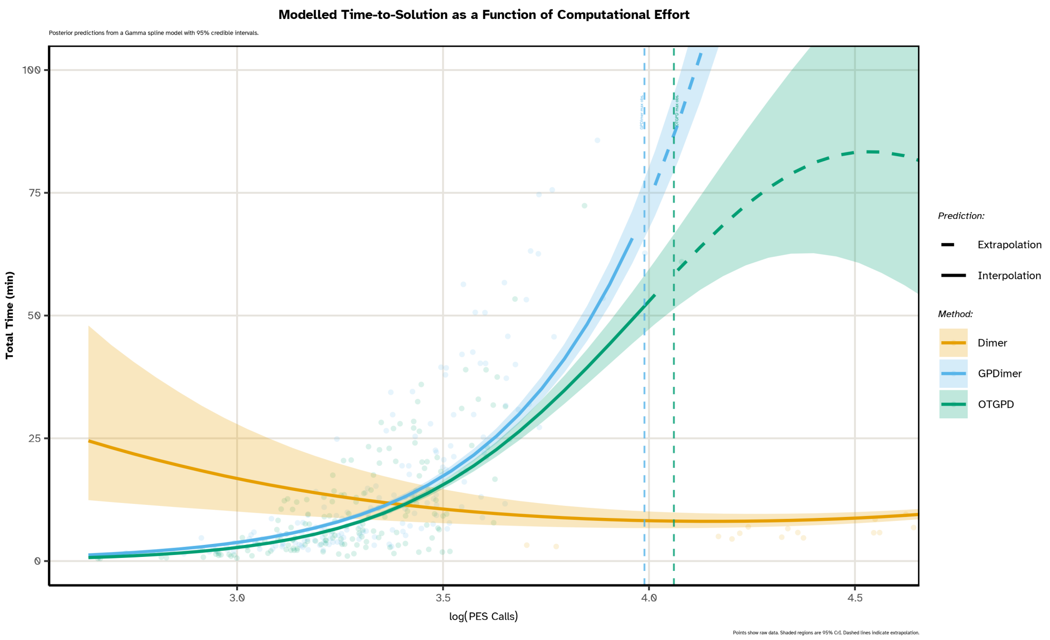

Figure S2: Posterior predictions from the hierarchical Gamma spline model for total wall-clock time as a function of PES evaluations. Points represent the raw data for each of the three methods. Solid lines are the model's posterior mean predictions, with shaded regions indicating the 95% credible intervals. The relationship between time and PES calls is allowed to be non-linear and is modeled independently for each method. For the GP-based methods, dashed lines indicate where the model is extrapolating beyond the maximum number of observed PES calls for that method, which are marked by the vertical dashed lines.

While this model provides a robust estimate of the median performance over the data seen, the full extent of OTGPD's superiority, particularly its ability to avoid the worst-case timings that affect GPDimer, is most clearly visualized by the performance profiles in the main text. The model shows that OTGPD reduces the median time-to-solution to less than a half compared to the dimer method (9.0 min vs 20.5 min) and runs $\approx$ 30% faster than the GPDimer (9.0 min vs 12.8 min), with a greater than 99% probability that OTGPD outperforms the other two methods.

S8 GPDimer / OTGPD failure modes

The OTGPD framework successfully eliminates the signal variance instability. 4 failures were observed in the OTGPD benchmark but not in the GPDimer which we consider in this section.

These were not due to an algorithmic instability but were artifacts of the dimer initialization routine.

For the OTGPD benchmark, initial dimer configurations were generated by displacing atoms along the softest mode found by an inexpensive xtb calculation. For systems D016, D084, D100, and S242, this procedure created a pathological starting geometry with excessively high forces or atoms in unphysically close contact.

When any GP-accelerated method is "poisoned" with such a high-energy, high-force baseline, the initial surrogate model learns that these unphysical configurations are normal, leading to an unstable search that fails to converge. The GPDimer benchmark, used a different (and in these cases, fortuitously non-pathological) initialization based on displacing the least coordinated atom, bypassed this specific problem. Figure S3 shows the initial points at the start of the algorithm runs, extracted from the trajectory files.

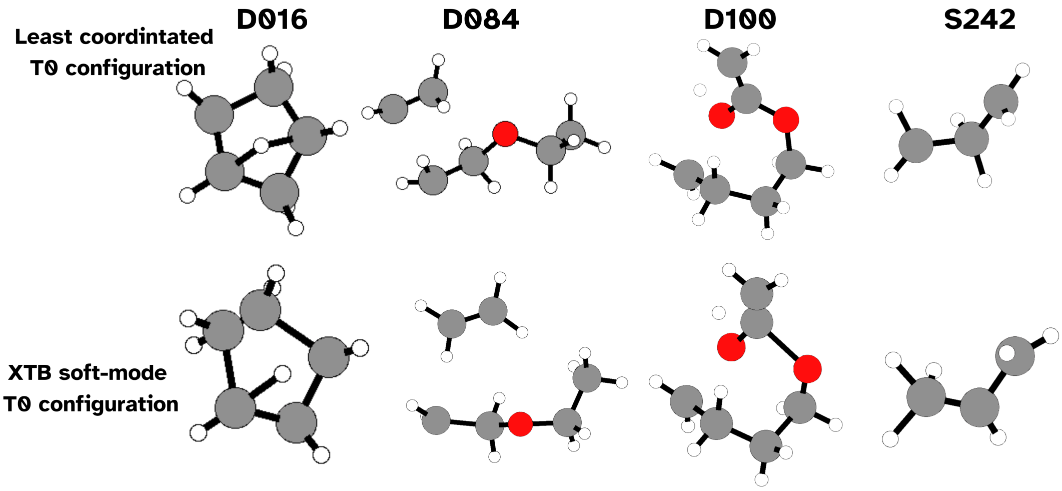

Figure S3: Initializations for the GPDimer (top) and OTGPD (bottom). The XTB initialization procedure in these four cases results in starting the runs from unphysical initial geometries leading to failures in the optimization routine. With XTB, D016 has unphysically close carbon atoms, D084 has a shortened carbon-oxygen bond, D100 has near-overlapping carbon atoms, and S242 has a CH3 end-group instead of the hydrogen on the middle carbon.

To confirm this diagnosis, we re-ran these four systems using OTGPD but starting from the exact same (valid) initial configurations used by the GPDimer runs to show all methods converge.

Times reported in minutes -> `total seconds / 60`. Calls represent the number of Hartree-Fock calculations, which are the "true energy samples" required.

S8.1 D016

Method	Calls	Time
Dimer	106	7.4
GPDimer	20	3.7
OTGPD	20	3.3

S8.2 D084

Method	Calls	Time
Dimer	2666	255.4
GPDimer	75	417
OTGPD	65	181.4

S8.3 D100

Method	Calls	Time
Dimer	214	24.3
GPDimer	28	16.2
OTGPD	28	18.52

S8.4 S242

Method	Calls	Time
Dimer	249	14.6
GPDimer	25	2.3
OTGPD	33	1.85

References

[1] S. T. Chill, M. Welborn, R. Terrell, L. Zhang, J.-C. Berthet, A. Pedersen, H. Jónsson, G. Henkelman, *Modelling and Simulation in Materials Science and Engineering* **2014**, *22*, 055002.

[2] E. Aprà, E. J. Bylaska, W. A. De Jong, N. Govind, K. Kowalski, T. P. Straatsma, M. Valiev, H. J. J. Van Dam, Y. Alexeev, J. Anchell, V. Anisimov, F. W. Aquino, R. Atta-Fynn, J. Autschbach, N. P. Bauman, J. C. Becca, D. E. Bernholdt, K. Bhaskaran-Nair, S. Bogatko, P. Borowski, J. Boschen, J. Brabec, A. Bruner, E. Cauët, Y. Chen, G. N. Chuev, C. J. Cramer, J. Daily, M. J. O. Deegan, T. H. Dunning, M. Dupuis, K. G. Dyall, G. I. Fann, S. A. Fischer, A. Fonari, H. Früchtl, L. Gagliardi, J. Garza, N. Gawande, S. Ghosh, K. Glaesemann, A. W. Götz, J. Hammond, V. Helms, E. D. Hermes, K. Hirao, S. Hirata, M. Jacquelin, L. Jensen, B. G. Johnson, H. Jónsson, R. A. Kendall, M. Klemm, R. Kobayashi, V. Konkov, S. Krishnamoorthy, M. Krishnan, Z. Lin, R. D. Lins, R. J. Littlefield, A. J. Logsdail, K. Lopata, W. Ma, A. V. Marenich, J. Martin Del Campo, D. Mejia-Rodriguez, J. E. Moore, J. M. Mullin, T. Nakajima, D. R. Nascimento, J. A. Nichols, P. J. Nichols, J. Nieplocha, A. Otero-de-la-Roza, B. Palmer, A. Panyala, T. Pirojsirikul, B. Peng, R. Peverati, J. Pittner, L. Pollack, R. M. Richard, P. Sadayappan, G. C. Schatz, W. A. Shelton, D. W. Silverstein, D. M. A. Smith, T. A. Soares, D. Song, M. Swart, H. L. Taylor, G. S. Thomas, V. Tipparaju, D. G. Truhlar, K. Tsemekhman, T. Van Voorhis, Á. Vázquez-Mayagoitia, P. Verma, O. Villa, A. Vishnu,

K. D. Vogiatzis, D. Wang, J. H. Weare, M. J. Williamson, T. L. Windus, K. Woliński, A. T. Wong, Q. Wu, C. Yang, Q. Yu, M. Zacharias, Z. Zhang, Y. Zhao, R. J. Harrison, *Journal of Chemical Physics* **2020**, *152*, 184102.
[3] O.-P. Koistinen, V. Ásgeirsson, A. Vehtari, H. Jónsson, *Journal of Chemical Theory and Computation* **2020**, *16*, 499.
[4] C. Bannwarth, S. Ehlert, S. Grimme, *Journal of Chemical Theory and Computation* **2019**, *15*, 1652.
[5] M. Gunde, N. Salles, A. Hémeryck, L. Martin-Samos, *Journal of Chemical Information and Modeling* **2021**, *61*, 5446.
[6] R. Goswami, *AIP Advances* **2025**, *15*, 85210.
